# Diversity and Biosynthetic Potential of Culturable Actinomycetes Associated with Marine Sponges in the China Seas

**DOI:** 10.3390/ijms13055917

**Published:** 2012-05-16

**Authors:** Lijun Xi, Jisheng Ruan, Ying Huang

**Affiliations:** State Key Laboratory of Microbial Resources, Institute of Microbiology, Chinese Academy of Sciences, Beijing 100101, China; E-Mails: xilijun1002@163.com (L.X.); jishengruan@yahoo.com.cn (J.R.)

**Keywords:** actinomycetes, marine sponge, diversity, biosynthetic potential, antimicrobial, PKS, NRPS, phylogenetic analysis

## Abstract

The diversity and secondary metabolite potential of culturable actinomycetes associated with eight different marine sponges collected from the South China Sea and the Yellow sea were investigated. A total of 327 strains were isolated and 108 representative isolates were selected for phylogenetic analysis. Ten families and 13 genera of *Actinomycetales* were detected, among which five genera represent first records isolated from marine sponges. Oligotrophic medium M5 (water agar) proved to be efficient for selective isolation, and “*Micromonospora*–*Streptomyces*” was proposed as the major distribution group of sponge-associated actinomycetes from the China Seas. Ten isolates are likely to represent novel species. Sponge *Hymeniacidon perleve* was found to contain the highest genus diversity (seven genera) of actinomycetes. Housekeeping gene phylogenetic analyses of the isolates indicated one ubiquitous *Micromonospora* species, one unique *Streptomyces* species and one unique *Verrucosispora* phylogroup. Of the isolates, 27.5% displayed antimicrobial activity, and 91% contained polyketide synthase and/or nonribosomal peptide synthetase genes, indicating that these isolates had a high potential to produce secondary metabolites. The isolates from sponge *Axinella* sp. contained the highest presence of both antimicrobial activity and NRPS genes, while those from isolation medium DNBA showed the highest presence of antimicrobial activity and PKS I genes.

## 1. Introduction

Marine sponges (Phylum *Porifera*) are multicellular invertebrate sessile filter-feeders that provide unique and favorable environmental conditions for microbial colonization and often harbor abundant and diverse microbes. Microbial communities associated with marine sponges are very complex, contributing up to 40% of the sponge biomass [1,2]. Marine sponge-associated bacterial communities include the following taxa: *Acidobacteria*, *Actinobacteria*, *Bacteroidetes*, *Chlamydiae*, *Chloroflexi*, *Cyanobacteria*, *Deinococcus-Thermus*, *Firmicutes*, *Gemmatimonadetes*, *Nitrospira*, *Planctomycetes*, *Proteobacteria*, *Spirochaetes* and *Verrucomicrobia* [3–7]. Among the bacterial associates, members of *Actinobacteria* are often sponge-specific [4,8] and have been identified as dominant producers of biologically active compounds [9–11]. There is evidence that the presence of biosynthesis genes encoding polyketide synthases (PKSs) and nonribosomal peptide synthetases (NRPSs) in marine sponge-derived actinomycetes are useful indicators for the selection of strains to isolate new natural products [12].

Actinomycetes are widely distributed in marine sponges. At the time of writing, over 30 sponge genera had been reported to be hosts of actinomycetes, with ten genera having each been collected in different sea areas [4,5,7,8,13–18]. Among the nearly 10,000 sponge-derived microbial sequences submitted to public databases, about one-sixth belong to *Actinobacteria* [19], indicating that this is an important group among sponge-associated microorganisms. Actinomycetes abundance in marine sponges is variable but can make up over 20% of the total microorganisms in some marine sponges [20,21]. The study of marine-sponge-associated actinobacterial diversity involves both culture-dependent and culture-independent methods. In the past decade, a large number of marine sponge-derived actinomycetes have been identified using culture methods, spanning 26 genera [12,22–29]. The use of culture-independent methods has enabled the detection of an additional five genera of actinomycetes in marine sponges, as well as many unculturable novel actinobacterial taxa [21,30,31]. Although both of the above-mentioned methods have defects and bias, the culture-dependent method is still popular even in the “omics” age [32]. This is partly because the isolates yielded from this method provide very useful phenotypic and genotypic information [33], such as physiological traits and biosynthetic potential, for further ecological investigation and bioprospecting.

Diverse sponges are found in the China Seas, with the South China Sea being estimated to contain nearly half of the marine sponge species in the world [34]. The aim of this study was to investigate the diversity and biosynthetic potential of culturable actinomycetes associated with various sponges from the South China Sea and the Yellow Sea. To this end, several selective isolation media were used, and the isolates were subjected to phylogenetic analyses based on 16S rRNA and other housekeeping genes, and were tested for antimicrobial activity as well as the presence of secondary metabolite genes encoding polyketide synthases (PKS I and PKS II) and nonribosomal peptide synthetases (NRPSs).

## 2. Results and Discussion

### 2.1. Selective Isolation

A detailed description of the eight sponge samples that were processed is listed in Table 1. A quantitative analysis on the efficiency of the six media for actinomycete isolation is shown in Table 2. The water agar (M5) medium indicated the best isolation effect in terms of both selectivity and yield (percentage and number of actinomycete colonies). High selectivity values, but much lower yields, were recorded in the dilute nutrient broth agar (DNBA) and oatmeal agar (ISP 3). A good isolation effect, with relatively high selectivity and yield, was noted in water agar with sea salt (M5-S), while mannitol-peptone agars (SMP and SMP-S) were not effective in terms of isolating actinomycetes from sponges in the China Seas.

The quantitative data of this study indicated that oligotrophic media were suitable for the culture and isolation of marine sponge-associated actinomycetes. Medium M5, containing only agar and water, was the most suitable, in that it demonstrated the highest selectivity and yield, and the second-highest diversity of isolates. This contrasts with previous reports which showed that media containing appropriate nutrient were suitable for isolating actinomyctes from marine sponges [8,23,24,27–29] and is the first account of oligotrophic media being suitable for this purpose. Moreover, supplementing the medium with sea salt could not help the isolation because both the selectivity and yield of M5-S and SMP-S decreased. This is also supported by the fact that none of the isolates obligately depend on sea salt, as they all grew well on the purification medium yeast extract-malt extract agar (ISP 2) or starch-yeast extract-peptone agar (M1).

Most colonies fall into two main groups, based on colony morphology: the *Micromonospora*-like group (tiny, colored, solid colonies with unusual aerial hyphae) and the *Streptomyces*-like group (large colonies with aerial spore mass). The relative abundance between these two groups is 9:2, indicating that the *Micromonospora*-like actinomycetes were dominant among the sponges. A total of 327 isolates were purified from the isolation plates, based on sample ID, culture medium and strain morphology. Nearly half (46%) of the isolates were from sponges *Axinyssa* sp. WNGB3 (26%) and *Xestospongia* sp. QYP07 (20%), while the sponge *Spongia* sp. LCJ-1 yielded the smallest number of isolates (5%) (Table 1).

### 2.2. Phylogenetic Analysis and Diversity

According to morphological characteristics and the source sponge, 108 representative isolates were selected. The nearly full-length (> 1300 bp) 16S rRNA genes of representative isolates were sequenced and subjected to phylogenetic analysis. Results showed that these belonged to ten families and 13 genera—*Bogoriellaceae* (*Georgenia*), *Geodermatophilaceae* (*Blastococcus*), *Micrococcaceae* (*Kocuria*), *Micromonosporaceae* (*Catenuloplanes*, *Micromonospora*, *Verrucosispora*), *Nocardiaceae* (*Gordonia*), *Nocardiopsaceae* (*Nocardiopsis*), *Pseudonocardineae* (*Pseudonocardia*, *Saccharomonospora*), *Streptomycetaceae* (*Streptomyces*), *Streptosporangiaceae* (*Nonomuraea*), and *Thermomonosporaceae* (*Actinomadura*). The relative abundances of isolates from these genera were as follows: *Micromonospora* (56 isolates: 51.9%), *Streptomyces* (30 isolates: 27.8%), *Verrucosispora* (7 isolates: 6.5%), *Nocardiopsis* (3 isolates: 2.8%), *Actinomadura* (2 isolates: 1.9%), *Catenuloplanes* (2 isolates: 1.9%), *Kocuria* (2 isolates: 1.9%), and *Gordonia*, *Georgenia*, *Blastococcus*, *Pseudonocardia*, *Nonomuraea*, and *Saccharomonospora* (each with one isolate and a relative abundance of 0.9%). Around 26 genera of culturable actinomycetes isolated from marine sponges have so far been reported. Our study added five genera to this record: *Actinomadura*, *Blastococcus*, *Catenuloplanes*, *Georgenia* and *Nonomuraea*.

The isolates formed 35, 25 and 19 OTUs, defined by 16S rRNA gene sequence distances of 0.01, 0.02 and 0.03, respectively. The highest sequence similarities to recognized species with valid names were as follows: 100% (23 isolates), 99.0–99.9% (75 isolates) and <99.0% (10 isolates). The latter ten isolates (Table 3) may represent novel species of actinomycetes, among which strain FXJ6.011 has been established as the type strain of *Micromonospora yangpuensis* sp. nov. [35].

**Figure 1:**
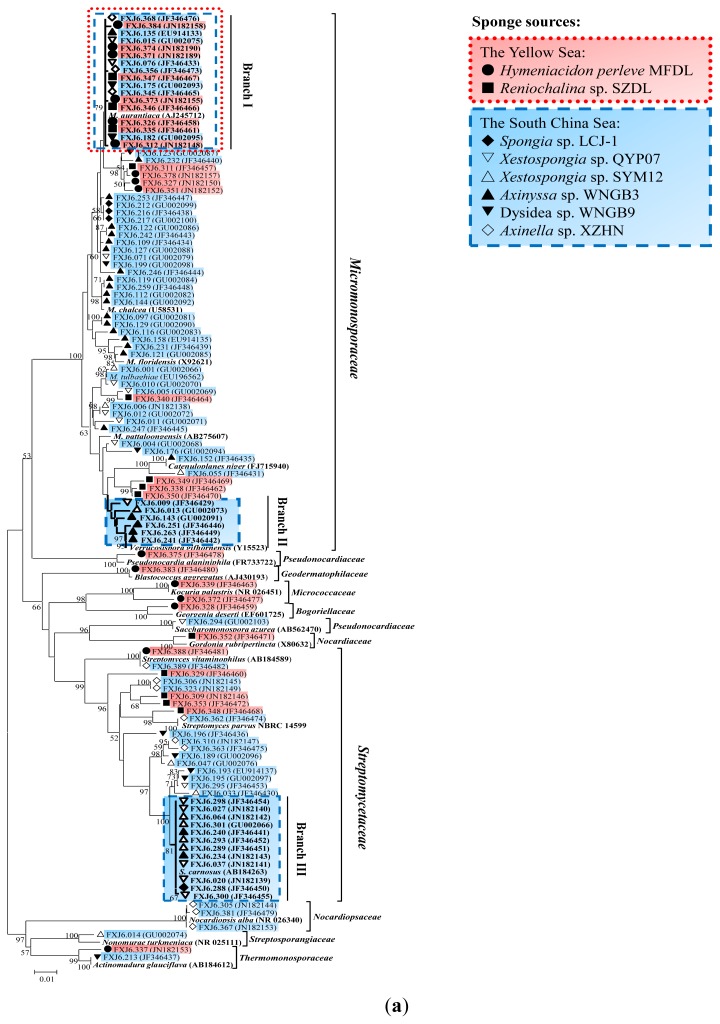

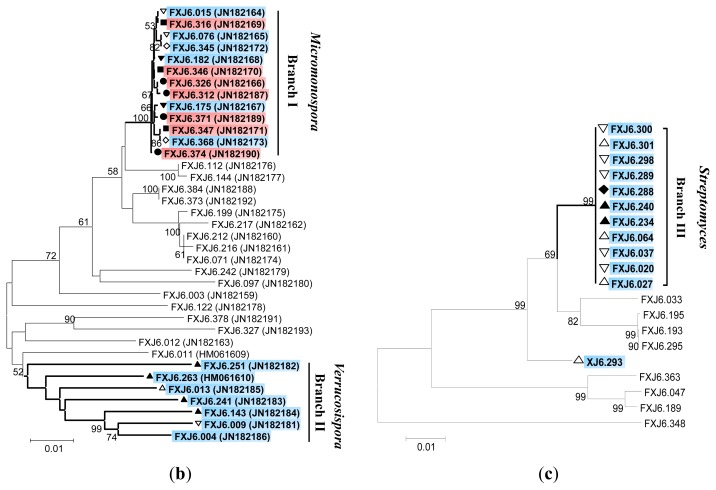
Neighbor-joining trees based on (**a**) 16S rRNA gene sequences (>1300bp) of 108 representative isolates and 19 related type strains of different genera; (**b**) *gyrB* gene sequences (1106 bp) of Branch I, II and relative isolates; and (**c**) *atpD*-*gyrB*-*recA*-*rpoB*-*trpB* concatenated sequences (>2500 bp) of Branch III and relative isolates. Percentage bootstrap values based on 1000 resampled data sets are shown at the nodes; only values above 50% are given. The scale bar indicates 0.01 nucleotide substitution per nucleotide position. Different sponge sources of isolates were marked with different symbols and colors.

The majority (60.2%) of isolates fell into the family *Micromonosporaceae* and formed diversiform branches in the 16S rRNA gene tree within three genera (Figure 1a): *Micromonospora*, *Verrucosispora* and *Catenuloplanes*. The second-most diverse isolates fell into the genus *Streptomyces*, with a diversity of 98.2–100% 16S rRNA gene sequence similarities. Three large phylogenetic branches were circumscribed in the genera *Micromonospora*, *Verrucosispora* and *Streptomyces*, each containing isolates from different sponges (Figure 1a). Although members of Branch I were isolated from the South China Sea and the Yellow Sea sponges, they indicated a close relationship to each other, with ≤0.001 16S rRNA gene (>1300 bp) distances and ≤0.003 *gyrB* gene (1106 bp) distances (Figure 1b). According to quantitative evidence on the relationship of 16S rRNA sequence similarity to DNA-DNA re-association in actinomycetes [36], as well as evidence indicating that the *gyrB*-based genetic distance of 0.014 would correspond to 70% DNA homology among *Micromonospora* species [37], the 13 isolates of this branch were identified as a single *Micromonospora* species, which is widely distributed in sponges of the two China Seas. Branch III contained 12 closely-related *Streptomyces* isolates that were only detected in the South China Sea sponges, with ≤0.001 16S rRNA gene sequence distances. With the exception of isolate FXJ6.293, the other 11 isolates shared identical 16S rRNA and multi-locus (*atpD*-*gyrB*-*recA*-*rpoB*-*trpB*, >2500 bp) sequences (Figure 1c), and therefore belonged to the same species [38,39]. In contrast, Branch II contained diverse members of the genus *Verrucosispora*, with 16S rRNA gene distances of 0.005–0.021 and the *gyrB* gene distances of 0.029–0.085. Members of this branch have only been isolated from the South China Sea sponges.

**Figure 2:**
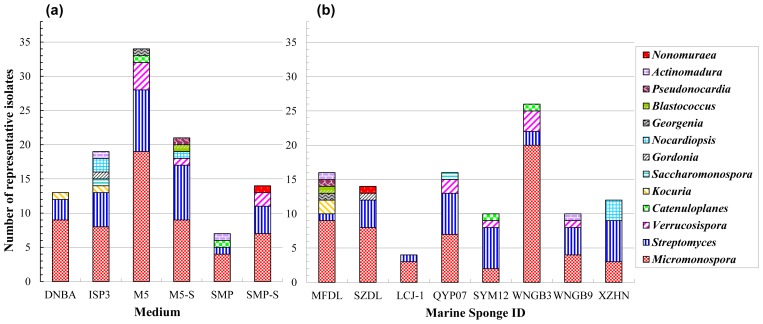
Numbers of representative isolates belonging to different genera, (**a**) recovered on six different selective media and; (**b**) isolated from eight sponge samples.

The media associated with isolate diversity are shown in Figure 2a. Oatmeal agar (ISP 3) yielded the highest diversity of isolates, being associated with seven genera; water agar (M5) yielded the largest number of isolates, being associated with five genera, while water agar with sea salt (M5-S) yielded obviously fewer isolates of six genera; and dilute nutrient broth agar (DNBA) yielded isolates of only three genera. Sponge-associated isolate diversity is shown in Figure 2b. The most diverse group of actinomycetes was collected from sponge *Hymeniacidon perleve* MFDL, which contained seven genera, but albeit accounted for only 8% of the isolates. This was followed by the sponge host *Xestospongia* sp. SYM12, to which five actinomycetes genera were associated, and four genera were associated with each of the following three sponges: *Xestospongia* sp. QYP07, *Axinyssa* sp. WNGB3 and *Dysidea* sp. WNGB9. Sponges *Axinella* sp. XZNH and *Reniochalina* sp. SZDL each harbored three genera, and only two genera were associated with sponge *Spongia* sp. LCJ-1. Actinomycetes genera *Micromonospora* and *Streptomyces* were recovered on all of the media and from each sponge sample.

Selvin *et al.* [8] found that the “*Micromonospora–Saccharomonospora–Streptomyces*” group is a major culturable actinobacterial group in the marine sponge *Dendrilla nigra*. According to our investigation, *Micromonospora* spp. and *Streptomyces* spp. can be isolated from all of the eight sponges, but *Saccharomonospora* sp. was only isolated from the sponge *Xestospongia* sp QYP07. In seven out of eight sponges, the *Micromonospora* colony was dominant, and *Streptomyces* was the dominant genus in the sponge *Axinella* sp. XZNH and the sub-dominant genus in the other seven sponges. Taking these results into consideration, we propose that “*Micromonospora–Streptomyces*” is the major group of culturable marine sponge-associated actinomycetes in the China Seas.

The family *Micromonosporaceae* is widely distributed in the sea and five genera of this family have been recorded from marine environments: *Asanoa*, *Micromonospora*, *Polymorphospora*, *Salinospora* and *Verrucosispora* [40–43], of which *Micromonospora*, *Salinospora* and *Verrucosispora* can be isolated from marine sponges. In this study, the isolates of the family *Micromonosporaceae* were found to be dominant, in terms of quantity and diversity, in most marine sponges, and three genera of this family were isolated as well. However, we did not find the genus *Salinospora*; instead, we obtained *Catenuloplanes*. This is the first report of the genus *Catenuloplanes* from marine sponges.

### 2.3. Antimicrobial Activity and Gene Screening

Two hundred and forty-four isolates (including the 108 representative isolates) were tested for antimicrobial activity and the presence of PKS I, PKS II and NRPS genes (Table 4). Sixty-seven tested isolates (27.5%) displayed antimicrobial activity, belonging to the genera *Streptomyces* (34 isolates), *Micromonospora* (30 isolates), *Actinomadura*, *Gordonia* and *Pseudonocardia* (supplementary Table S1). Isolates associated with sponge *Axinella* sp. XZHN (mostly *Streptomyces* spp.) had the highest occurrence of antimicrobial activity (58.6%) compared with those of other sponge-associated isolates, notably the activity against *Candidia albicans* (31%). Of the tested isolates, 222 (91%) contained PKS/NRPS genes, and isolates associated with sponge *Dysidea* sp. WNGB9 showed the highest occurrence of PKS/NRPS genes (100%). The average detection rates of PKS I, PKS II and NRPS genes of the isolates were 54.9, 58.6, 60.2%, respectively, and the highest rates were detected in isolates from sponges *Spongia* sp. LCJ-1 (75.0%), *Xestospongia* sp. SYM12 (92.0%) and *Axinella* sp. XZHN (77.8%). Isolates from sponge *Axinella* sp. XZHN showed the highest presence of both antimicrobial activity and NRPS genes, indicating that they may possess good biosynthetic potential; while isolates from sponge *Reniochalina* sp. SZDL showed the lowest presence of both antimicrobial activity (16.1%) and PKS/NRPS genes (80.6%). It is also noticeable that isolates from dilute nutrient broth agar (DNBA) showed the highest presence of antimicrobial activity as well as PKS I gene (supplementary Table S2), despite the fact that this medium gave low yield and the genus diversity of the isolates.

Results from our study indicate that the actinomycetes from China Sea sponges have higher, or equal, percentages of antimicrobial activity as well as the presence of biosynthetic genes, in comparison with isolates derived from other marine environments [12,44,45]. It was shown that strains in which either PKS or NRPS genes were identified produced a significantly higher number of metabolites and exhibited a larger number of unidentified metabolites than other strains [12]. Most organisms (91%) isolated in our study have the potential to produce secondary metabolites. Although only seven out of the 67 bioactive isolates were against *E. coli*, this is not frustrating because Gram-negative bacteria are generally more resistant to antibiotics due to their unique outer membrane, and we used only one medium (ISP 2 or M1) to cultivate the isolates for antimicrobial assay.

There is evidence that sponges have the capacity to “assemble” associated microorganisms, which would be useful for their survival [46–48]. Apart from rare pathogenic strains, actinomycetes are generally not harmful and can produce abundant bioactive metabolites [10,12,15,25,49], e.g., the widespread *Micromonospora* strains are not only useful in biomedicine, biocontrol and potentially in biofuels [50], but also closely related with nitrogen fixation in plant root nodules [51]. This might be the major reason for the assembly of actinomycetes in marine sponges: for the protection and benefit of the sponge host. The identification of bioactive small molecules and gaining an understanding of their roles in the interactions between sponges and symbiotic actinomycetes will become a major focus of future studies.

## 3. Experimental Section

### 3.1. Sampling

Six specimens of the marine sponges were collected from the South China Sea and two specimens from the Yellow Sea, by means of SCUBA diving to depths of up to 10 m. Samples were kept on ice in fresh seawater and later transported to the laboratory where they were stored at −20 °C.

### 3.2. Selective Isolation and Dereplication of Actinomycetes

To remove transient and loosely attached bacteria, each sponge sample was thoroughly washed at least 5 times with sterile water until clear, after which sponge material of 1 g was cut into pieces measuring approximately 1 cm^3^ and then homogenized in sterile mortars. Homogenates were heated in a water bath at 55 °C for 6 min, diluted in series, and plated in triplicate on agar plates. Based on the previous researches and pre-experiments, six media were used for isolation: oatmeal agar (ISP 3; DSMZ medium 609), mannitol-peptone agar (SMP) and mannitol-peptone agar with sea salt (SMP-S) [52], water agar (M5) and water agar with sea salt (M5-S) [53], and dilute nutrient broth agar (DNBA) [54]. All of the media were supplemented with nalidixic acid and nystatin (each at 25 mg·L^−1^). The inoculated plates were incubated at 28 °C for 30–60 days. Colonies were counted and representative colonies from each sample were picked out and purified on yeast extract-malt extract agar (ISP 2; DSMZ medium 65) or starch-yeast extract-peptone agar (M1) [53] plates. The isolates were divided into different groups according to a number of morphological characteristics, including color of aerial and substrate mycelia, diffusible pigments, arrangement of hyphae and spore chains, and spore shape.

### 3.3. Antimicrobial Activity Screening

An agar diffusion assay was used for antimicrobial screening against indicator microorganisms, including bacteria *Bacillus subtilis* CGMCC 1.2428 (=DSM 347), *Escherichia coli* CGMCC 1.2385 (=DSM 1103) and *Staphylococcus aureus* subsp. *aureus* CGMCC 1.2386 (=DSM 1104), as well as yeast *Candidia albicans* CGMCC 2.538. Agar plugs that were fully covered with a mass of isolated strains were cut from agar plates (ISP 2 or M1 media) that were incubated at 28 °C for 14 days, and transferred to test plates containing individual indicator strains, after which they were incubated at either 28 °C or 37 °C for 12–24 h. Diameters of the inhibition zone were measured for the purpose of describing the antimicrobial activity.

### 3.4. DNA Extraction, 16S rRNA and Housekeeping Gene Amplification

Extraction of genomic DNA, PCR amplification and sequencing of the 16S rRNA gene were performed, following the methods described by Chun and Goodfellow [55]. The methods used for PCR amplification of the *gyrB* (DNA gyrase B subunit) gene and sequencing of the PCR products in members of the family *Micromonosporaceae* are outlined in Garcia *et al.* [56]. For *Streptomyces* strains, five housekeeping genes—*atpD* (ATP synthase F1, β-subunit), *gyrB*, *recA* (recombinase A), *rpoB* (RNA polymerase, β-subunit) and *trpB* (tryptophan synthase, β-subunit)—were amplified and sequenced according to the methods developed by Guo *et al.* [57] and Rong *et al.* [58].

### 3.5. Phylogenetic Analysis

The resultant 16S rRNA gene (>1300 bp) and *gyrB* gene (>1100 bp) sequences were compared with those deposited in the public databases using the NCBI BLAST program and were aligned with related sequences retrieved from the public databases using the Clustal W algorithm in MEGA version 5.0 [59]. The gene sequence similarity values were calculated after pairwise alignment using MEGA version 5.0. The five housekeeping gene sequences (*ca*. 500 bp each) of the isolates were aligned and trimmed manually at the same position, prior to multi-locus sequence analysis, and concatenated by joining head-to-tail in-frame in the following order: *atpD-gyrB-recA-rpoB-trpB* (>2500 bp). Phylogenetic trees were constructed in MEGA version 5.0 using the neighbor-joining method [60]. Evolutionary distance matrices were calculated with the Kimura 2-parameter model. The Bootstrap test [61] was used to evaluate the reliability of the inferred tree, based on 1000 replications.

### 3.6. Detection of NRPS and PKS Genes

To assess the genetic potential of the isolates for producing bioactive secondary metabolites, the amplification of genes encoding polyketide synthases (PKS I and PKS II) and non-ribosomal peptide synthetase (NRPS) from the isolates was carried out using degenerate primers recommended by Ayuso-Sacido & Genilloud [62] and Metsa-Ketela *et al.* [63]. The PCR amplification reagents and programs were based on those of Rong *et al.* [38].

### 3.7. Nucleotide Sequence Accession Numbers

The 16S rRNA gene sequences of representative isolates were deposited in GenBank database under the following accession numbers: EU914133, EU914135, EU914137, GU002066, GU002068-GU002076, GU002079, GU002081-GU002088, GU002090-GU002100, GU002103, JF346429-JF346431, JF346433-JF346482 and JN182138-JN182158. GenBank accession numbers for the partial *gyrB* sequences of *Micromonospora* and *Verrucosispora* isolates were JN182159-JN182194, and those for the housekeeping genes of *Streptomyces* isolates were JQ258943-JQ259047.

## 4. Conclusions

This research demonstrates the rich diversity of culturable actinomycetes from marine sponges in the South China Sea and the Yellow sea, with different, and similar, population structures. Five genera represent first records associated with marine sponges, and ten isolates are likely to represent novel species. Oligotrophic medium seems to be efficient for selective isolation. Antimicrobial activity and PKS/NRPS gene screening indicated that, with “*Micromonospora–Streptomyces*” as the major group, the sponge-associated actinomycetes from the China Seas have high biosynthetic potential, and could serve as a good resource for the exploration of bioactive natural products.

## Supplementary Materials



## Figures and Tables

**Table 1:** Detailed accounts of the sponge samples collected from the Yellow Sea and the South China Sea.

	Sample ID	Sponge Species	Location	Collection Time	No. of Isolates (%)
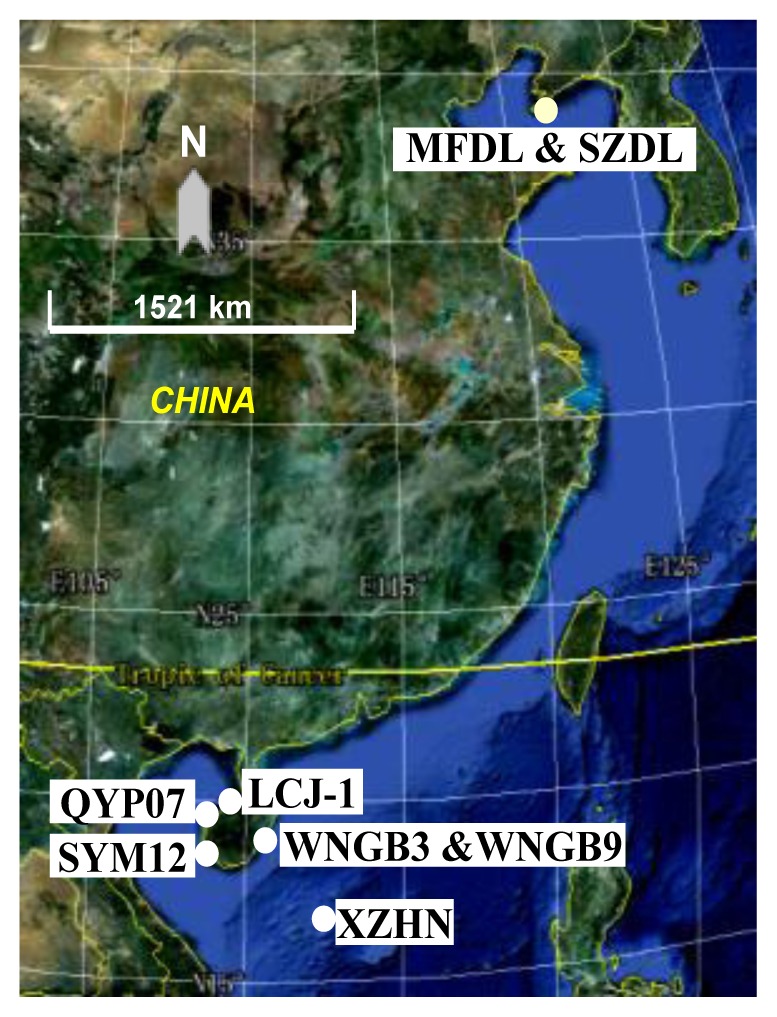	MFDL	*Hymeniacidon perleve*	YS, N38°51.497 E121°32.357	2009.03	26 (8)
	SZDL	*Reniochalina* sp.	YS, N38°51.497 E121°32.357	2009.03	35 (11)
	LCJ-1	*Spongia* sp.	SCS, N19°55.269 E109°29.118	2006.04	15 (5)
	QYP07	*Xestospongia* sp.	SCS, N19°39.925 E109°04.678	2006.04	65 (20)
	SYM12	*Xestospongia* sp.	SCS, N18°19.478 E108°57.229	2006.03	44 (13)
	WNGB3	*Axinyssa* sp.	SCS, N18°53.068 E110°31.314	2006.04	85 (26)
	WNGB9	*Dysidea* sp.	SCS, N18°53.068 E110°31.314	2006.04	27 (8)
	XZHN	*Axinella* sp.	SCS, N16°83 E121°33	2009.03	30 (9)

YS, Yellow Sea; SCS, South China Sea.

**Table 2:** Colony numbers of actinomycetes and non-actinomycetes recorded on six different selective media seeded with all eight samples.

Medium	Total No. (%) of *Actinomycetes*	Total No. (%) of Other Microbes	No. of *Actinomycetes* per Plate
Dilute nutrient broth agar (DNBA)	427 (96)	17 (4)	9
Oatmeal agar (ISP 3)	126 (93)	9 (7)	3
Water agar (M5)	2076 (96)	83 (4)	44
Water agar with sea salt (M5-S)	1318 (90)	152 (10)	28
Mannitol-peptone agar (SMP)	1712 (45)	2105 (55)	36
Mannitol-peptone agar with sea salt (SMP-S)	690 (10)	6189 (90)	15

**Table 3:** The ten isolates having <99% 16S rRNA gene sequence similarities with the closest species.

Isolate	GenBank No.	Closest Species	Type strain’s GenBank No.	Similarity (%)	Isolation Medium	Source Sponge
FXJ6.011	GU002071	*Micromonospora chaiyaphunensis*	AB196710	98.7	SMP-S	*Xestospongia* sp. QYP07
FXJ6.013	GU002073	*Verrucosispora sediminis*	EU870859	98.6	SMP-S	*Xestospongia* sp. SYM12
FXJ6.014	GU002074	*Nonomuraea turkmeniaca*	NR_025111	98.2	M5	*Xestospongia* sp. SYM12
FXJ6.251	JF346446	*Verrucosispora gifhornensis*	AB546292	98.8	M5	*Axinyssa* sp. WNGB3
FXJ6.309	JN182146	*Streptomyces sclerotialus*	AB184071	98.2	M5-S	*Hymeniacidon perleve* MFDL
FXJ6.328	JF346459	*Georgenia muralis*	AB455495	96.8	M5	*Hymeniacidon perleve* MFDL
FXJ6.338	JF346462	*Micromonospora pattaloongensis*	AB275607	98.9	ISP 3	*Reniochalina* sp. SZDL
FXJ6.348	JF346468	*Streptomyces laceyi*	AB249944	98.9	DNBA	*Reniochalina* sp. SZDL
FXJ6.349	JF346469	*Micromonospora pattaloongensis*	AB275607	97.8	ISP 3	*Reniochalina* sp. SZDL
FXJ6.350	JF346470	*Micromonospora pattaloongensis*	AB275607	98.9	DNBA	*Reniochalina* sp. SZDL

**Table 4:** Numbers of positive isolates for antimicrobial activity and PKS I, PKS II and NRPS genes (percentage/tested isolates) from different sponges.

Character	MFDL	SZDL	LCJ-1	QYP07	SYM12	WNGB3	WNGB9	XZNH	Average
Anti-*B. subtilis*	21.1/19	16.1/31	18.2/11	6.5/31	33.3/15	15.3/85	8.7/23	34.5/29	17.6/244
Anti-*C. albicans*	21.1/19	6.5/31	0/11	9.7/31	6.7/15	1.2/85	17.4/23	31.0/29	9.8/244
Anti-*E. coli*	5.3/19	0/31	9.1/11	3.1/31	0/15	2.4/85	4.3/23	3.4/29	2.9/244
Anti-*S. aureus*	0/19	3.2/31	9.1/11	9.7/31	0/15	7.1/85	13.0/23	3.4/29	6.1/244
Activity *	31.6/19	16.1/31	27.3/11	19.4/31	33.3/15	20.0/85	34.8/23	58.6/29	27.5/244

PKS I	61.9/21	41.9/31	75.0/12	62.2/37	56.0/25	48.6/74	64.7/17	59.3/27	54.9/244
PKS II	57.1/21	48.4/31	41.7/12	75.7/37	92.0/25	51.4/74	76.5/17	33.7/27	58.6/244
NRPS	61.9/21	32.3/31	50.0/12	62.2/37	68.0/25	59.5/74	76.5/17	77.8/27	60.2/244
PKS/NRPS	90.5/21	80.6/31	91.7/12	94.6/37	96.0/25	90.5/74	100/17	96.3/27	91/244

* At least against one indicator microorganism.
