# Supplementary material for: Diversity and Biosynthetic Potential of Culturable Actinomycetes Associated with Marine Sponges in the China Seas
Source: Int J Mol Sci. 2012 May 16;13(5):5917–32. doi: 10.3390/ijms13055917 (PMC3382808; doi:10.3390/ijms13055917)
Supplement: Supplementary file 1 [file ijms-13-05917-s001.pdf]

Supplementary Materials

Supplementary Table S1. Numbers of positive isolates for antimicrobial activity and PKS I, PKS II and NRPS genes (percentage/tested isolates) from different isolation media.

**Character**	**M5**	**M5-S**	**ISP3**	**DNBA**	**SMP**	**SMP-S**
Anti-*B. subtilis*	17.0/88	25.6/43	13.2/38	28.6/28	7.7/13	9.7/31
Anti-*C. albicans*	4.5/88	9.3/43	13.2/38	28.6/28	7.7/13	12.9/31
Anti-*E. coli*	3.4/88	2.3/43	0/38	7.1/28	0/13	3.2/31
Anti-*S. aureus*	5.8/88	4.7/43	2.6/38	14.3/28	7.7/13	16.1/31
Activity *	25.0/88	32.6/43	21.1/38	42.9/28	15.4/13	29.0/31
PKS I	58.0/88	46.9/49	56.1/41	80.6/31	50.0/8	55.6/27
PKS II	59.0/88	57.1/49	56.1/41	61.3/31	50.0/8	66.7/27
NRPS	59.0/88	59.2/49	61.0/41	58.1/31	87.5/8	66.7/27
PKS/NRPS	90.9/88	89.8/49	92.7/41	93.5/31	87.5/8	96.3/27

* At least against one indicator microorganism.

Supplementary Table S2. Details of the 67 bioactive isolates isolated from different sponge types.

Isolate No.	Genus	Source Sponge	PKS I	PKS II	NRPS	*E. coli*	*S. aureus*	*C. albicans*	*B. subtilis*	Medium
FXJ6.309	*Streptomyces*	*Hymeniacidon perleve* MFDL	-	-	+	-	-	-	+	M5-S
FXJ6.372	*Micromonospora*	*Hymeniacidon perleve* MFDL	+	-	+	+	-		+	DNBA
FXJ6.373	*Micromonospora*	*Hymeniacidon perleve* MFDL	+	-	+	-	-	+	+	DNBA
FXJ6.374	*Micromonospora*	*Hymeniacidon perleve* MFDL	+	+	-	-	-	+	+	DNBA
FXJ6.378	*Micromonospora*	*Hymeniacidon perleve* MFDL	-	+	+	-	-	+	-	DNBA
FXJ6.387 *	*Micromonospora*	*Hymeniacidon perleve* MFDL	-	-	-	-	-	+	-	M5-S
FXJ6.311	*Streptomyces*	*Reniochalina* sp. SZDL	-	+	-	-	-	-	+	ISP3
FXJ6.314	*Micromonospora*	*Reniochalina* sp. SZDL	+	+	-	-	-	-	+	M5-S
FXJ6.317	*Micromonospora*	*Reniochalina* sp. SZDL	-	+	-	-	-	-	+	M5-S
FXJ6.353	*Gordonia*	*Reniochalina* sp. SZDL	+	-	+	-	+	+	+	M5
FXJ6.376	*Micromonospora*	*Reniochalina* sp. SZDL	-	+	+	-	-	+	+	M5-S
FXJ6.206	*Micromonospora*	*Spongia* sp. LCJ-1	+	-	+	-	+	-	-	M5-S
FXJ6.208	*Micromonospora*	*Spongia* sp. LCJ-1	-	+	+	+	-	-	+	M5
FXJ6.286	*Micromonospora*	*Spongia* sp. LCJ-1	+	-	+	-	-	-	+	M5
FXJ6.002	*Micromonospora*	*Xestospongia* sp. QYP07	-	+	+	-	-	+	-	M5
FXJ6.017	*Micromonospora*	*Xestospongia* sp. QYP07	-	+	-	-	-	+	-	ISP3
FXJ6.049	*Streptomyces*	*Xestospongia* sp. QYP07	-	+	-	-	+	-	-	SMP
FXJ6.052	*Streptomyces*	*Xestospongia* sp. QYP07	-	+	+	+	+	+	+	M5-S
FXJ6.301	*Streptomyces*	*Xestospongia* sp. QYP07	+	+	+	-	-	-	+	M5
FXJ6.296	*Streptomyces*	*Xestospongia* sp. QYP07	+	+	+	-	-	-	+	SMP-S
FXJ6.297	*Streptomyces*	*Xestospongia* sp. QYP07	+	+	-	-	+	-	-	M5
FXJ6.008	*Micromonospora*	*Xestospongia* sp. SYM12	-	+	+	-	-	-	+	M5
FXJ6.288	*Streptomyces*	*Xestospongia* sp. SYM12	+	+	+	-	-	-	+	DNBA
FXJ6.289	*Streptomyces*	*Xestospongia* sp. SYM12	-	+	-	-	-	-	+	M5
FXJ6.001 *	*Micromonospora*	*Xestospongia* sp. SYM12	-	-	-	-	-	+	+	SMP
FXJ6.101	*Micromonospora*	*Axinyssa* sp. WNGB3	+	+	+	+	+	-	-	SMP-S
FXJ6.104	*Streptomyces*	*Axinyssa* sp. WNGB3	+	+	+	+	-	-	-	M5
FXJ6.106	*Micromonospora*	*Axinyssa* sp. WNGB3	+	+	-	-	-	-	+	M5

Supplementary Table S2. *Cont.*

FXJ6.116	*Micromonospora*	*Axinyssa* sp. WNGB3	+	-	+	-	+	-	-	M5
FXJ6.233	*Micromonospora*	*Axinyssa* sp. WNGB3	-	-	+	-	-	-	+	ISP3
FXJ6.240	*Streptomyces*	*Axinyssa* sp. WNGB3	-	-	+	-	+	-	+	ISP3
FXJ6.242	*Micromonospora*	*Axinyssa* sp. WNGB3	+	+	+	-	+	-	+	M5
FXJ6.247	*Micromonospora*	*Axinyssa* sp. WNGB3	-	+	+	-	+	-	+	DNBA
FXJ6.248	*Micromonospora*	*Axinyssa* sp. WNGB3	+	-	-	-	+	-	+	DNBA
FXJ6.250	*Micromonospora*	*Axinyssa* sp. WNGB3	+	-	+	-	-	-	+	M5
FXJ6.252	*Micromonospora*	*Axinyssa* sp. WNGB3	+	+	-	-	-	-	+	M5
FXJ6.260	*Micromonospora*	*Axinyssa* sp. WNGB3	+	+	+	-	-	-	+	M5
FXJ6.263	*Micromonospora*	*Axinyssa* sp. WNGB3	-	+	+	-	-	-	+	M5
FXJ6.264	*Micromonospora*	*Axinyssa* sp. WNGB3	+	-	+	-	-	+	-	M5
FXJ6.275	*Micromonospora*	*Axinyssa* sp. WNGB3	+	-	+	-	-	-	+	M5
FXJ6.284	*Streptomyces*	*Axinyssa* sp. WNGB3	-	+	+	-	-	-	+	M5
FXJ6.285	*Streptomyces*	*Axinyssa* sp. WNGB3	+	-	+	-	-	-	+	M5
FXJ6.172 *	*Streptomyces*	*Dysidea* sp. WNGB9	-	-	-	+	-	-	-	M5
FXJ6.192	*Micromonospora*	*Dysidea* sp. WNGB9	-	-	+	-	-	+	-	SMP-S
FXJ6.193	*Streptomyces*	*Dysidea* sp. WNGB9	-	-	+	-	-	+	-	SMP-S
FXJ6.194	*Streptomyces*	*Dysidea* sp. WNGB9	-	-	+	-	-	+	-	SMP-S
FXJ6.196	*Streptomyces*	*Dysidea* sp. WNGB9	-	-	+	-	+	-	+	SMP-S
FXJ6.197	*Streptomyces*	*Dysidea* sp. WNGB9	+	+	+	-	+	-	+	SMP-S
FXJ6.204	*Streptomyces*	*Dysidea* sp. WNGB9	+	-	-	-	-	+	-	SMP-S
FXJ6.213	*Actinomadura*	*Dysidea* sp. WNGB9	+	+	-	-	+	-	-	SMP-S
FXJ6.305	*Streptomyces*	*Axinella sp.* XZHN	+	-	+	-	-	+	-	M5-S
FXJ6.306	*Streptomyces*	*Axinella sp.* XZHN	+	-	-	-	-		+	M5-S
FXJ6.308	*Streptomyces*	*Axinella sp.* XZHN	+	+	+	-	-	-	+	M5-S
FXJ6.310	*Streptomyces*	*Axinella sp.* XZHN	+	-	+	-	-	-	+	M5-S
FXJ6.322	*Streptomyces*	*Axinella sp.* XZHN	+	+	+	-	-	-	+	M5-S
FXJ6.323	*Streptomyces*	*Axinella sp.* XZHN	+	+	+	-	-	-	+	M5-S

Supplementary Table S2. *Cont.*

FXJ6.343 *	*Streptomyces*	*Axinella sp.* XZHN	-	-	-	-	+	-	-	DNBA
FXJ6.358	*Streptomyces*	*Axinella sp.* XZHN	-	+	+	-	-	+	-	DNBA
FXJ6.359	*Streptomyces*	*Axinella sp.* XZHN	+	+	+	-	-	+	-	M5
FXJ6.362	*Micromonospora*	*Axinella sp.* XZHN	+	+	+	-	-	+	-	ISP3
FXJ6.363	*Streptomyces*	*Axinella sp.* XZHN	+	+	-	-	-	-	+	M5-S
FXJ6.364	*Streptomyces*	*Axinella sp.* XZHN	+	-	+	+	-	-	-	DNBA
FXJ6.365	*Streptomyces*	*Axinella sp.* XZHN	-	-	+	-	-	+	+	DNBA
FXJ6.366	*Streptomyces*	*Axinella sp.* XZHN	-	-	+	-	-	+	-	ISP3
FXJ6.368	*Streptomyces*	*Axinella sp.* XZHN	+	+	+	-	-	+	+	DNBA
FXJ6.370	*Streptomyces*	*Axinella sp.* XZHN	-	+	+	-	-	+	+	ISP3
FXJ6.381	*Pseudonocardia*	*Axinella sp.* XZHN	+	-	-	-	-	+	+	ISP3

* No PKS/NPRS genes were detected in this study.

© 2012 by the authors; licensee MDPI, Basel, Switzerland. This article is an open access article distributed under the terms and conditions of the Creative Commons Attribution license (http://creativecommons.org/licenses/by/3.0/).
